# Predicting Endurance Time in a Repetitive Lift and Carry Task Using Linear Mixed Models

**DOI:** 10.1371/journal.pone.0158418

**Published:** 2016-07-05

**Authors:** Ben Beck, Daniel J. Ham, Stuart A. Best, Greg L. Carstairs, Robert J. Savage, Lahn Straney, Joanne N. Caldwell

**Affiliations:** 1 Land Division, Defence Science and Technology Group, Fishermans Bend, Australia; 2 Department of Epidemiology and Preventive Medicine, Monash University, Melbourne, Australia; 3 Centre for Human and Applied Physiology, Faculty of Health and Behavioural Sciences, University of Wollongong, Wollongong, Australia; Florida International University Herbert Wertheim College of Medicine, UNITED STATES

## Abstract

**Objectives:**

Repetitive manual handling tasks account for a substantial portion of work-related injuries. However, few studies report endurance time in repetitive manual handling tasks. Consequently, there is little guidance to inform expected work time for repetitive manual handling tasks. We aimed to investigate endurance time and oxygen consumption of a repetitive lift and carry task using linear mixed models.

**Methods:**

Fourteen male soldiers (age 22.4 ± 4.5 yrs, height 1.78 ± 0.04 m, body mass 76.3 ± 10.1 kg) conducted four assessment sessions that consisted of one maximal box lifting session and three lift and carry sessions. The relationships between carry mass (range 17.5–37.5 kg) and the duration of carry, and carry mass and oxygen consumption, were assessed using linear mixed models with random effects to account for between-subject variation.

**Results:**

Results demonstrated that endurance time was inversely associated with carry mass (R^2^ = 0.24), with significant individual-level variation (R^2^ = 0.85). Normalising carry mass to performance in a maximal box lifting test improved the prediction of endurance time (R^2^ = 0.40). Oxygen consumption presented relative to total mass (body mass, external load and carried mass) was not significantly related to lift and carry mass (β_1_ = 0.16, SE = 0.10, 95%CI: -0.04, 0.36, p = 0.12), indicating that there was no change in oxygen consumption relative to total mass with increasing lift and carry mass.

**Conclusion:**

Practically, these data can be used to guide work-rest schedules and provide insight into methods assessing the physical capacity of workers conducting repetitive manual handling tasks.

## Introduction

Repetitive manual handling tasks are a significant source of work-related musculoskeletal injury [1–3]. To date, strategies to reduce injury in manual handling tasks have focussed on developing appropriate work-rest schedules [4] and designing tasks to match the physical capacity of the worker population [5].

Current guidance for repetitive manual handling tasks predominately stems from studies investigating: comfortable lifting limits during an 8-hour work day [6,7], changes in kinematics over time [8,9] and spinal loading [10,11]. However, only two studies have reported time to fatigue, or endurance time, for a repetitive manual handling task [12,13]. These studies demonstrated a clear relationship between endurance time and lifting rate or lifted mass. However, they are limited by an inability to explain individual variation in their repeated measures study designs. Previous research has demonstrated that utilising linear mixed models can be used to account for individual variation [14,15]. To our knowledge, there is only one study that has previously investigated endurance time in a lift and carry task [16]. These authors investigated the relationship between a unilateral team stretcher carry and multiple bilateral jerry can carries [16]. However, the purpose of that study was to develop an appropriate physical assessment of stretcher carriage and hence individual variation in time to fatigue was not relevant to the outcomes of this research. Additionally, previous guidelines on repetitive manual handling tasks commonly report a single standard for given task parameters that is designed to be applied across a diverse working population [6,7,17]. As such, individual variation is not accounted for in these previous work-related guidelines.

Given the reported injury risks associated with repetitive lift and carry tasks [18], there is clear need to investigate endurance time in repetitive manual handling tasks and understand variation at the individual-level. This data could be used to guide task design, set work-rest schedules and develop appropriate fitness assessments for repetitive lift and carry tasks, thereby ensuring employees have the physical capacity to safely and successfully complete the work. The aim of this study was to investigate endurance time in a repetitive lift and carry task. Specifically, we aimed to understand the relationship between the mass of the item lifted and carried (including when this mass was normalised to individual maximal lifting performance) and both task duration and the physiological burden (i.e. oxygen consumption), using linear mixed models.

## Methods

### Subjects

Fourteen Australian Defence Force (ADF) male soldiers with no existing injuries volunteered to participate in the study (age 22.4 ± 4.5 yrs, height 1.78 ± 0.04 m, body mass 76.3 ± 10.1 kg). Thirty soldiers were invited to participate from the Royal Australia Corps of Signals. Sixteen soldiers either declined to participate or could not participate due to existing injury. For all assessments, participants wore disruptive pattern combat uniform including boots and body armour (10.9 kg), hip webbing (8.0 kg), and a replica F88 Austeyr weapon (4.6 kg); a total external mass of 23.5 kg. Each subject completed a medical clearance and gave written informed consent prior to participation. The study was approved by the Australian Defence Human Research Ethics Committee (Protocol 491–07).

### Study Design

Participants attended four assessment sessions that consisted of one lifting session and three lift and carry sessions of various object masses. Each assessment session was conducted to volitional fatigue and separated by at least 24 hours rest. The maximal lift task, box lift and place, was conducted on the first day. For the lift and carry tasks, 3 masses were chosen randomly within the physical capacity range of the individual, as defined by pilot testing (range 17.5–37.5 kg). The pilot testing involved individuals performing a lift and carry task with incrementing object mass until volitional fatigue and was conducted at least 48 hours prior to the four assessment sessions. During the assessment sessions, not all participants could attend each testing session resulting in some missing data (n = 5 missing data points); as such, there are a total of 37 data points. This data has previously been reported in abstract form [19].

### Box Lift and Place

The box lift and place (BLP) assessment required participants to start by squatting down and lifting a box (Trimcast Rotomoulders Pty. Ltd, 0.35 x 0.35 x 0.35 m, metal handles at 0.20 m from base) with an under hand grip to knuckle height, followed by a pause, then taking one step forward, another pause, and then lifting the box onto a 1.50-m platform. A correct lift required at least half of the box to be placed onto the platform without sliding or pushing the mass onto the platform tray. The initial mass of the box was 10 kg and increased by 5 kg after every successful lift. Participants performed this task to a maximal lifting effort. The last successful lift, maintaining correct technique, was recorded as their maximum lifting capacity. Participants who failed to complete a lift were allowed a second attempt at the same mass. If the second lift was unsuccessful, 2.5 kg was removed and the participant was granted another opportunity to complete the lift at the decreased mass. At least three minutes of rest was given between each lifting attempt.

### Lift and Carry Task

The lift and carry task was based on a simulation of repetitive loading of ammunition. The assessment involved lifting a replica 120-mm ammunition shell (PVC piping, 800 mm length, 100 mm diameter) from a cradle (height 0.5 m), carrying it for 10 m in front of the body. The replica shell was then lifted and placed into a second cradle (height 1.7 m), after which the participants walked 8 m without the shell. The entire sequence of lift-carry-lift and walk was performed in 20 s, thus, three lifts were performed each minute. This simulation was completed to a cadence where an audio cue was provided every 10 s to indicate the pace to the participants, to ensure that they were moving at 1 m.s^-1^; the first tone to indicate when to pick up the shell and the next to indicate when to place the shell into the cradle. Participants completed this task three times differing only in the mass carried. Object mass was varied between 17.5 and 37.5 kg. The point of failure was either self-determined from the participant when they could no longer maintain the cadence, or by the experimenters who deemed the lift unsuccessful through incorrect or unsafe lifting technique.

### Oxygen Consumption

Oxygen consumption measures (Metamax 3B, Cortex, Leipzig, Germany) were collected on all participants during each lift and carry task. Data were recorded at 5-s intervals. Oxygen consumption values represent the average of the second last minute of each task. Oxygen consumption is presented as absolute values (L.min^-1^), relative to body mass (mL.kg^-1^.min^-1^) and relative to total mass (mL.kg^-1^.min^-1^). Total mass is defined as the sum of body mass, torso-borne external mass and carried mass.

### Statistical Methods

The relationships between carry mass and the duration of carry, and carry mass and oxygen consumption, were assessed using linear mixed models with random effects to account for between-subject variation [14,15]. The strength of linear mixed models is their ability to model individual variation (random effects on the slope and intercept of individual linear regression) and can also account for an uneven number of observations per participant. A log-transformation of time was used as the primary outcome to ensure normality of residuals. Data are presented as median (range). Eq 1 depicts the relationship between duration and carry mass with a constant β_0_, random intercept u_0j_, slope β_1_, random slope u_1j_, and error ε_ij_.

$$Ln{\left(duration\right)}_{ij}=\;\beta_{0}+\;\beta_{1}carrymass_{ij}+\;u_{0j}\;+u_{1j}carrymass_{ij}+\;\epsilon_{ij}$$ (1)

Where *i* represents observation 1,2,..*i* and *j* represents participant 1,2,…,*j*.

A likelihood ratio test was used to compare the linear mixed models with and without a random slope on the carry mass. Models investigating the duration of carry were run using two dependent variables: absolute lift and carry mass and this mass represented as a percentage of maximal BLP mass (total of four models). Models investigating oxygen consumption were run using three dependent variables: absolute oxygen consumption, oxygen consumption normalised to body mass only, and normalised to total mass (total of three models). For each of these models, the relationship between the independent and dependent variables is described using the coefficient of determination. All analyses were conducted in Stata (Stata Statistical Software: Release 13. College Station, TX: StataCorp LP). Significance was set at an alpha level of 0.05.

## Results

Median time to fatigue (or endurance time) of the lift and carry task was 16:10 min (range: 2:55–125:20 min). When conducting the discrete maximal lifting task (a box lift and place) participants lifted an average mass of 42.7 ± 7.6 kg (range: 27.5–55.0 kg).

### Duration of Lift and Carry Task

The likelihood ratio test demonstrated that the addition of a random slope did not significantly improve model performance either in the model of absolute lift and carry mass (χ^2^ = 0.00, p = 1.00) nor in the model using mass relative to maximal BLP mass (χ^2^ = 0.05, p = 0.82). This demonstrates that the rate of decline in carry duration with increasing carry mass was consistent across all participants. Herein, the models are presented with a random intercept only.

**Fig 1:**
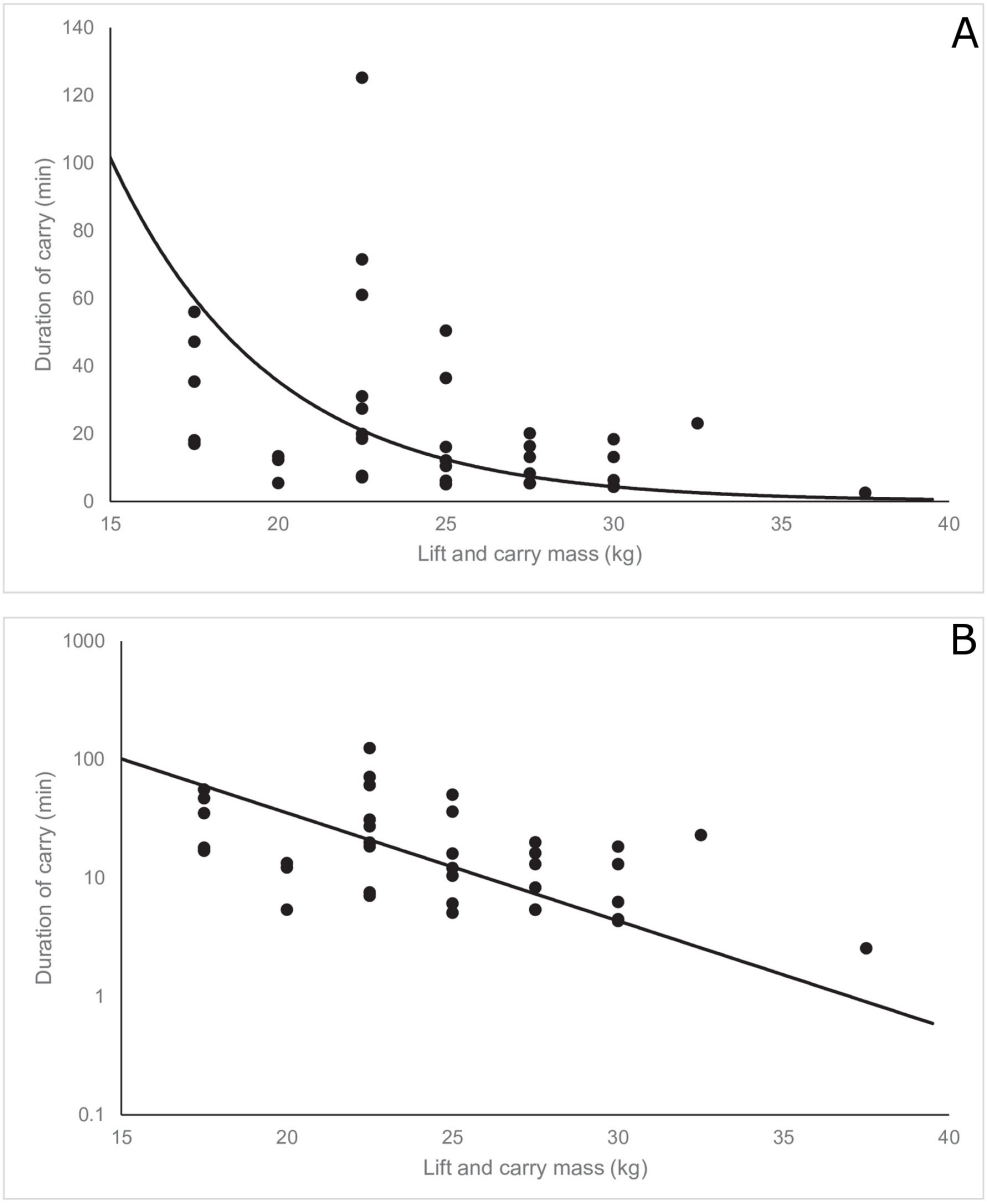
Plot of the duration of carry against lift and carry mass (n = 37) depicted without (A) and with (B) a logarithmic scale. The solid line depicts the prediction of the fixed portion of the linear mixed model.

**Fig 2:**
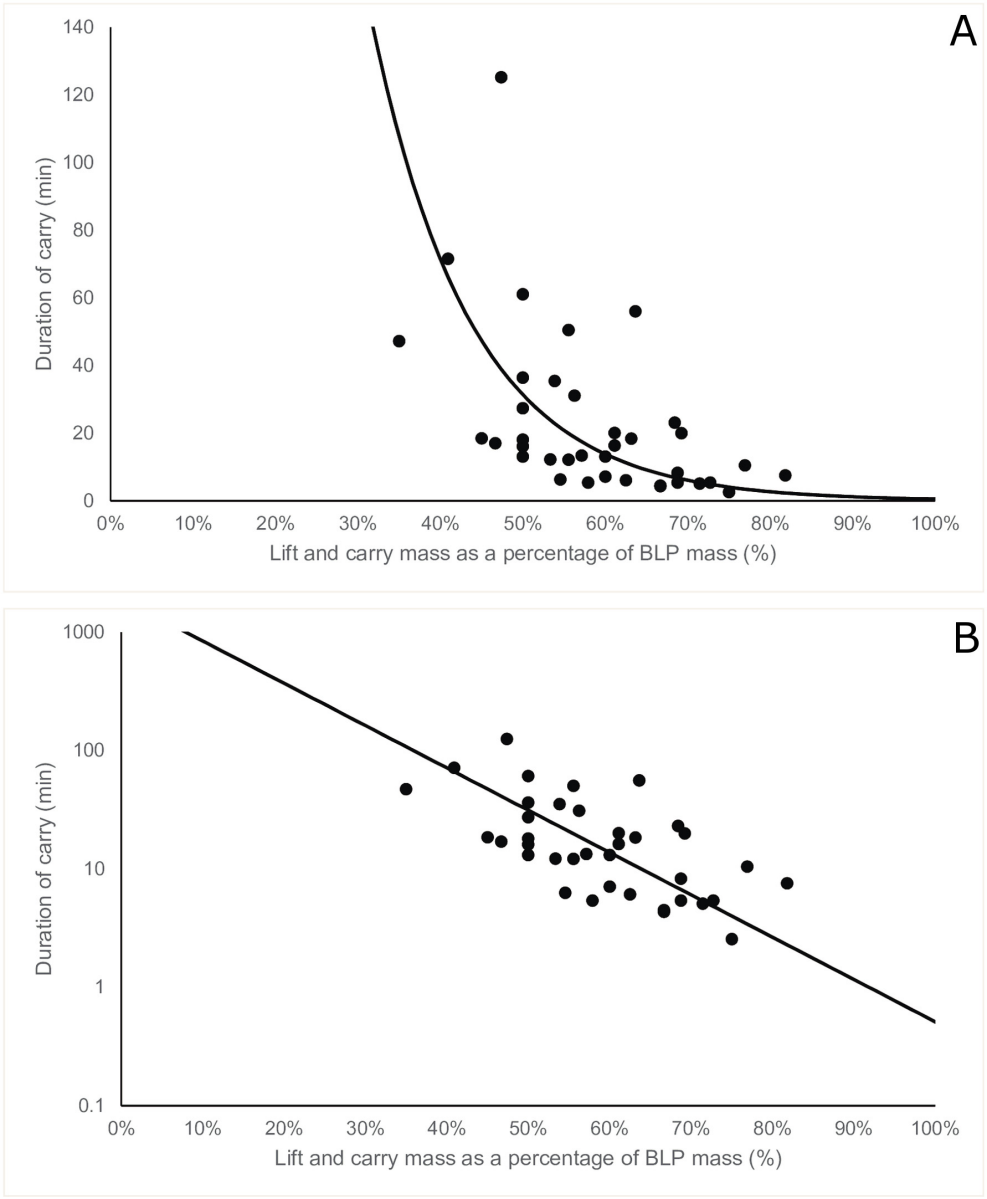
Plot of the duration of carry against lift and carry mass as a percentage of box lift and place (BLP) mass (n = 37) depicted without (A) and with (B) a logarithmic scale. The solid line depicts the prediction of the fixed portion of the linear mixed model.

The log-transformed duration of carry significantly declined with increasing absolute carry mass (β_1_ = -0.21, 95%CI: -0.25, -0.16, p<0.001, Fig 1) and increasing carry mass relative to maximal BLP mass (β_1_ = -8.23, 95%CI: -10.23, -6.23, p<0.001, Fig 2). Table 1 presents a summary of model outputs.

**Table 1:** Results of the linear mixed model predicting natural-log of carry duration. 95% CI denotes confidence intervals.

Lift and carry mass	Carry mass coefficient (standard error)	Constant (standard error)	Variance of level-two errors (SE)	Variance of residuals (SE)
Absolute	-0.21 (0.02)	7.77 (0.60)	0.62 (0.29)	0.20 (0.06)
Relative to BLP*	-8.23 (1.02)	7.56 (0.62)	0.33 (0.19)	0.24 (0.08)

* BLP denotes box lift and place.

Correlation coefficients from the linear predictions with contributions from random effects (range: R^2^ = 0.81–0.85) were greater than equivalent correlation coefficients using the fixed portion of the model only (Table 2). This indicates that the accuracy of predicting the duration of carry is greater when subject-specific information is used; that is, when you have at least one existing data point. Additionally, correlation coefficients using the fixed portion of the model improved with relative mass as the dependent variable (R^2^ = 0.40) compared to absolute mass (R^2^ = 0.24). There was less between-subject variation, measured by the variance of the random intercept, when accounting for relative mass versus absolute mass; Var(u_0j_ = 0.62) versus Var(u_0j_ = 0.33) for relative and absolute mass, respectively (Table 1).

**Table 2:** Correlation coefficients for the prediction of the natural log of duration. Coefficients are segregated by models with and without random effects. BLP denotes box lift and place.

Lift and carry mass	Coefficient of determination: fixed portion of model	Coefficient of determination: with random effects
Absolute	0.24	0.85
Relative to BLP	0.40	0.81

### Oxygen Consumption

**Fig 3:**
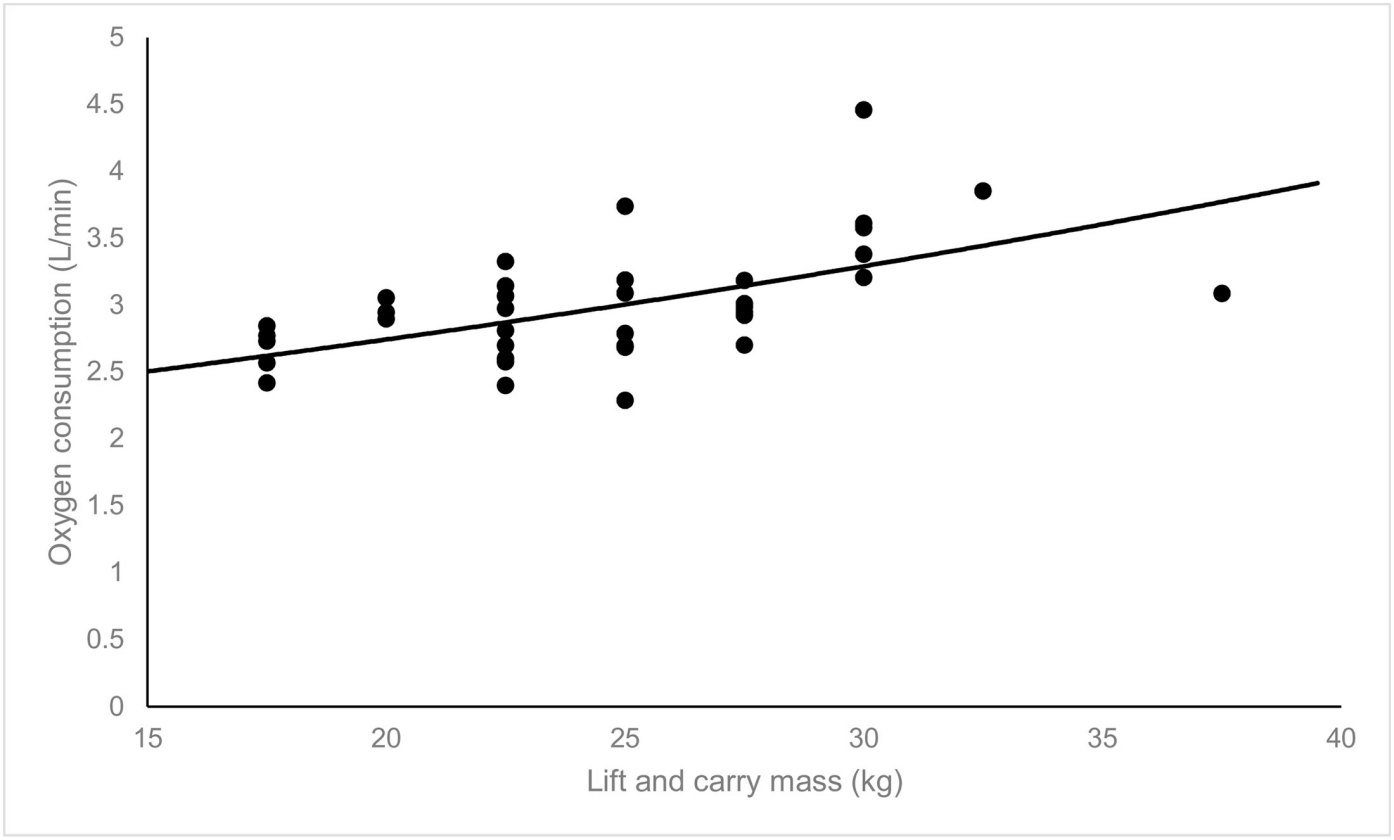
Plot of absolute oxygen consumption and lift and carry mass (n = 37). The solid line depicts the linear prediction of the fixed portion of the linear mixed model.

**Fig 4:**
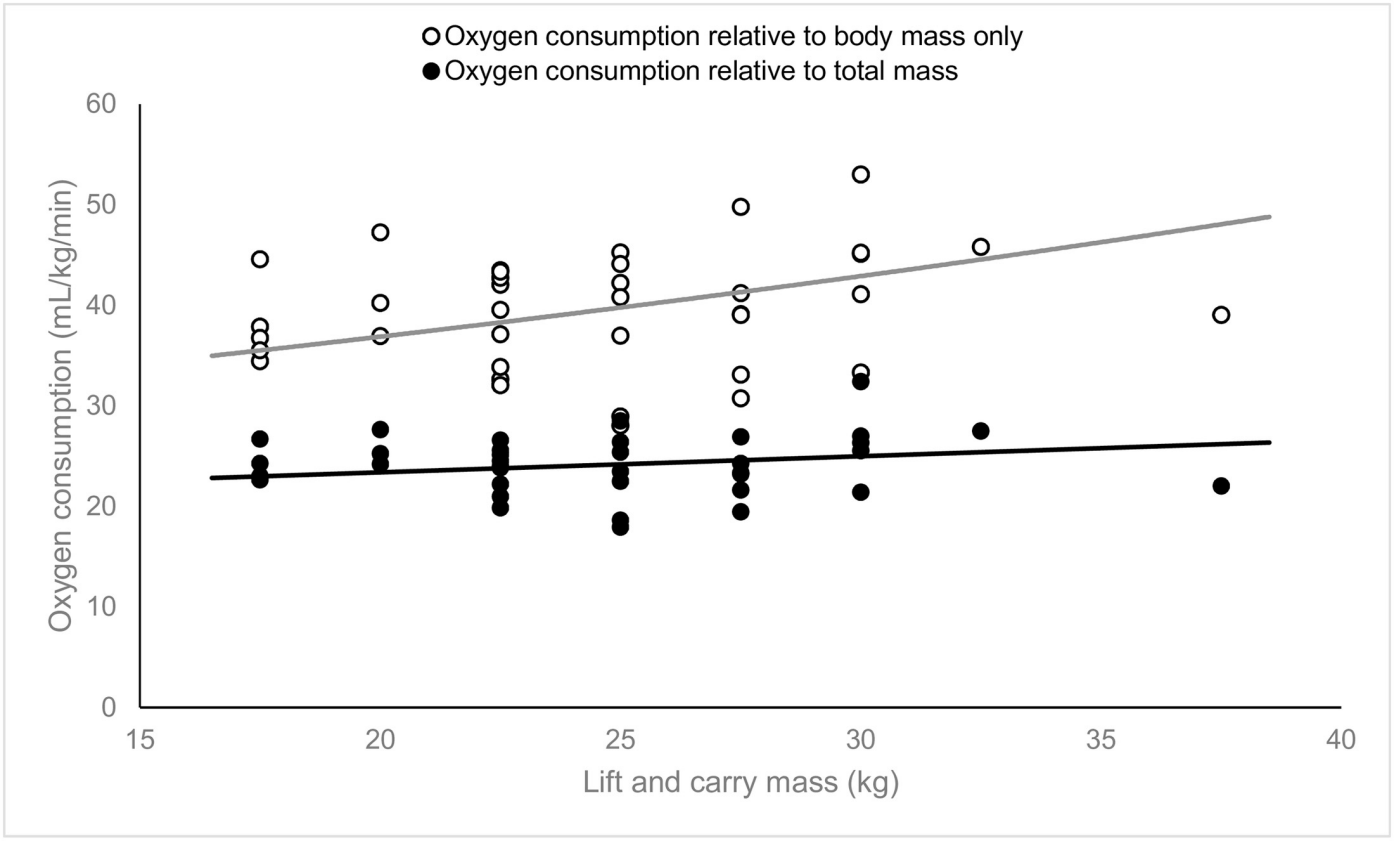
Plot of oxygen consumption relative to total mass (body mass + carried mass) (n = 37) and relative to body mass against lift and carry mass (n = 37). The solid lines depict the linear prediction of the fixed portion of the linear mixed model.

Absolute oxygen consumption increased significantly with increasing lift and carry mass (Fig 3); there was a 0.06 L.min^-1^ increase in oxygen consumption for each additional kilogram carried (β_1_ = 0.06, SE = 0.01, 95%CI: 0.03, 0.08, p<0.001). Similarly, oxygen consumption presented relative to body mass increased significantly with increasing lift and carry mass; there was a 0.6 mL.kg^-1^.min^-1^ increase in oxygen consumption relative to body mass for each additional kilogram carried (β_1_ = 0.61, SE = 0.18, 95%CI: 0.25, 0.96, p = 0.001). In contrast, oxygen consumption presented relative to total mass (body mass, external load and carried mass) was not significantly related to lift and carry mass (β_1_ = 0.16, SE = 0.10, 95%CI: -0.04, 0.36, p = 0.12), indicating that there was no change in oxygen consumption relative to total mass with increasing lift and carry mass. A comparison of the relationships between lift and carry mass and oxygen consumption relative to body mass and relative to carry mass is shown in Fig 4.

## Discussion

This is the first study to investigate the relationship between carry mass and endurance time in a repetitive lift and carry task. In this study, we showed that despite variation in absolute endurance time between individuals, the rate of decline in endurance time with increasing lift and carry mass was consistent between individuals. By normalising lift and carry mass to performance on a discrete maximal lifting task, the accuracy of the prediction equation was improved. Furthermore, variations in oxygen consumption were solely the result of variations in lift and carry mass. Together, these data provide important information regarding endurance time in a repeated lift and carry task and will find application in occupational settings.

Compared to existing studies investigating endurance time in repetitive manual handling tasks [12,13,20], the strength of our study lies in the ability to understand individual variation through the use of linear mixed models. Our results indicate that the decline in lift and carry duration with increasing carry mass was consistent at the individual level. In contrast to the model with the random intercept, the linear prediction of lift and carry duration using the fixed portion of the model only (the ‘overall’ regression) demonstrated lower correlation coefficients. This finding is unsurprising, since it is well accepted that individual variation in physical capacity substantially impacts endurance performance. This result also indicates that we can very accurately predict an individual’s carry duration across a range of carry masses if we have one existing data point from that individual. Practically, this means that you could assess an individual’s carry duration with a carry mass of 20 kg and then be able to accurately predict their carry duration at a carry mass of 30 kg. Furthermore, it would be possible to determine an equivalent work time for a lift and carry task of known duration if the mass of item was altered. As such, the developed equations would be beneficial to employers in determining appropriate work schedules under altered task conditions.

To further understand this individual variation, we controlled for a component of individual-level physical capacity by representing the lift and carry mass as a percentage of maximal lifting performance in the BLP. The results demonstrated a stronger correlation from the fixed portion of the model relative to absolute lift and carry mass. This indicates that a large component of the individual-level variation in endurance time is explained by maximal lifting performance. Given that muscular strength is strongly associated with maximal lift performance [21,22], this suggests that muscular strength plays a critical role in performance of the repetitive lift and carry task investigated in this study. Further work is required to understand the specific mechanisms limiting performance in this repetitive lift and carry task. Normalising the lift and carry mass to a maximal lifting task also allows a relationship to be developed between a lifting task and the duration of the lift and carry task, similar to that previously shown for a repetitive lifting task [20]. From our study, a maximal box lift and place can be used to predict duration of a lift and carry task for a given mass. It is important to note, however, that this prediction only explains 40% of the variation in the duration of carry. As described previously, predicting lift and carry duration is far more accurate (R^2^ = 0.85 vs R^2^ = 0.40) when one existing data point from an individual on the mass-duration curve is known.

This finding is critical when considering applying guidelines around repetitive manual handling tasks. The majority of published recommendations are designed to be implemented at a population-level [6,7,17]; for example, across an entire workforce. While this method facilitates an ease of implementation, it negates the individual-level variation in performance. We have previously demonstrated individual variation in a repetitive carrying task [16,23], suggesting that population-level guidelines may not be appropriate across a workforce. Given the results of this study demonstrate that accounting for individual variation substantially increases the explained variation in a repetitive lift and carry task, it is likely that accounting for such individual-level variation would be of significant benefit to employers in establishing guidelines on the conduct of repetitive manual handling tasks. Such a method could lead to tailored work-rest schedules for individuals based on their physical capacity and subsequently may lead to enhancements in workplace productivity. It is, however, important to note that we have demonstrated this for a single repetitive lift and carry task and further research is required to expand this method to other repetitive manual handling tasks.

Comparisons to existing studies must be made with caution as variations in task parameters exist. These studies have focussed on the impact of lifted mass and lift frequency on endurance time in repetitive lifting tasks [12,13]. Similar to the results of our study, endurance time was shown to have a strong negative relationship with increasing lifted mass. However, given the addition of a carry component in the task investigated in our study, direct comparisons between the results of our study and these previous studies are inappropriate. As we did not investigate the impact of lift frequency and platform height on endurance time, further work is required to understand these factors in repetitive lift and carry tasks.

To our knowledge, only one study has previously investigated changes in oxygen consumption associated with variations in the object mass of maximal repetitive manual handling tasks [13]. In this prior study, both absolute oxygen consumption and oxygen consumption normalised to body mass increased with lifted mass and lift frequency in a repetitive lifting task. Results reported in the repetitive lift and carry task investigated in our study showed a similar trend with increasing oxygen consumption (absolute and relative to body mass) with increasing lift and carry mass. However, our results extend this existing work by demonstrating that there was no change in oxygen consumption when normalised to total mass. Physiologically, this outcome was not unexpected since it is well known that increases in oxygen consumption are proportional to increasing load [24]. However, this is an important result as it highlights that within the manual handling task investigated in this study, changes in oxygen consumption are reliant on subject and item mass only. Existing manual handling studies report oxygen consumption as absolute or relative to body mass and, as a result, may not control for variations in individual task parameters. As such, by normalising oxygen consumption to total mass, more meaningful comparisons of physiological burden could be made between different manual handling tasks. Further work is required to determine whether this holds true for other repetitive manual handling tasks.

One of the limitations of this study is that these results apply specifically to the lift and carry task investigated and the range of carry masses used. Therefore, future research should be conducted to evaluate the utility of this model in other lift and carry tasks. Additionally, variations in lift frequency were not accounted for in this study and should be considered in future research. Our study used a cohort of male soldiers and therefore the results of this study may not be generalizable to the civilian workforce.

The implications of these results are two-fold; firstly these data can be used to understand endurance time on lift and carry tasks and set appropriate work-rest tables, and secondly, we provide evidence for the use of a maximal lifting task to aid in predicting endurance time. Work-rest schedules are used in a variety of occupations to guide task duration depending on factors such as sleep loss [25] and heat stress [26]. By understanding individual variation in the time to exhaustion in repetitive tasks, the models that we report can be used to guide the development of specific work-rest tables in lift and carry tasks or the effect a change in item mass may make to the sustainable work time of a worker population or specific worker. The linear mixed models also enable the accurate prediction of endurance time if one existing data point is known. In situations where time constraints may prevent an employer assessing an employee’s performance on a long-duration repetitive lift and carry task, these predictive models can be used to generate an equivalent shorter-duration task with increased lift and carry mass.

## Conclusions

This study investigated the endurance time and physiological demands of a repetitive lift and carry task. We observed that endurance time was inversely correlated with lift and carry mass, for which the decline was consistent between individuals. Prediction of endurance time improved when the lift and carry mass was expressed as a percentage of an individual’s maximal lifting capacity. Physiological measures revealed that variations in oxygen consumption were only due to variations in the combined individual and carried mass. The results of this study can be used to guide work-rest schedules in repetitive manual handling tasks and also provide insight into methods for assessing the physical capacity of workers conducting these tasks.
